# Revisiting adverse effects of cross-hybridization in Affymetrix gene expression data: do they matter for correlation analysis?

**DOI:** 10.1186/1745-6150-2-28

**Published:** 2007-11-07

**Authors:** Lev Klebanov, Linlin Chen, Andrei Yakovlev

**Affiliations:** 1 Department of Biostatistics and Computational Biology, University of Rochester, 601 Elmwood Avenue, Rochester, Box 630, New York 14642, USA; 2 Department of Probability and Statistics, Charles University, Sokolovska 83, Praha-8, CZ-18675, Czech Republic

## Abstract

**Background.:**

This work was undertaken in response to a recently published paper by Okoniewski and Miller (BMC Bioinformatics 2006, **7**: Article 276). The authors of that paper came to the conclusion that the process of multiple targeting in short oligonucleotide microarrays induces spurious correlations and this effect may deteriorate the inference on correlation coefficients. The design of their study and supporting simulations cast serious doubt upon the validity of this conclusion. The work by Okoniewski and Miller drove us to revisit the issue by means of experimentation with biological data and probabilistic modeling of cross-hybridization effects.

**Results.:**

We have identified two serious flaws in the study by Okoniewski and Miller: (1) The data used in their paper are not amenable to correlation analysis; (2) The proposed simulation model is inadequate for studying the effects of cross-hybridization. Using two other data sets, we have shown that removing multiply targeted probe sets does not lead to a shift in the histogram of sample correlation coefficients towards smaller values. A more realistic approach to mathematical modeling of cross-hybridization demonstrates that this process is by far more complex than the simplistic model considered by the authors. A diversity of correlation effects (such as the induction of positive or negative correlations) caused by cross-hybridization can be expected in theory but there are natural limitations on the ability to provide quantitative insights into such effects due to the fact that they are not directly observable.

**Conclusion.:**

The proposed stochastic model is instrumental in studying general regularities in hybridization interaction between probe sets in microarray data. As the problem stands now, there is no compelling reason to believe that multiple targeting causes a large-scale effect on the correlation structure of Affymetrix gene expression data. Our analysis suggests that the observed long-range correlations in microarray data are of a biological nature rather than a technological flaw.

**Reviewers:**

The paper was reviewed by I. K. Jordan, D. P. Gaile (nominated by E. Koonin), and W. Huber (nominated by S. Dudoit).

## 1. Background

Okoniewski and Miller [1] reported evidence they believe to be in favor of the idea that spurious positive correlations induced by the process of multiple targeting, i.e. the competition of multiple probe sets for a common transcript, represent a mass phenomenon in high-density oligonucleotide microarrays. They consider this phenomenon as a serious handicap to the inference on correlations in gene expression data analysis. In a way, their conclusion was in conflict with our re-analysis [2] of the Microarray Quality Control (MAQC) data [3] indicating that the level of technical noise in the contemporary Affymetrix platform is quite low. For this reason, we did not expect the effects of multiple targeting (MT) to be very disturbing. In [2], we argued as follows: "Since the competition of different oligonucleotide probes for the same transcript is random in nature, this process is expected to ultimately manifest itself in the observed technical variability, the latter having proven to be low. However, the proposed rationale is purely heuristic and cannot be independently verified as no technical vehicle is currently available for this purpose." This dissenting opinion drove us to look more closely at the problem from experimental and theoretical perspectives.

Another reason why we were unprepared to accept the conclusion by Okoniewski and Miller was that the proportion of problematic pairs of probe sets (among all pairs) was expected to be low because only their non-overlapping pairs should be considered. This point is discussed more elaborately in Section 2.1. We carried out the study reported in Section 2.1 to dispel our doubts. In doing so, our focus was on the prevalence of MT, and not on its significance in individual gene pairs. The latter problem, and especially its multiple testing aspect, is much more challenging from the statistical standpoint. Useful methodological results on significance of changes in correlation coefficients can be found in [4]. It is also beyond the scope of the present paper to discuss the potentially adverse effects of cross-hybridization on the outcomes of testing for differential expression. While such effects are plausible, we have no tools to investigate them quantitatively. At the same time, the publication by Okoniewski and Miller motivated us to provide a more in-depth analysis of the process of cross-hybridization based on the stochastic modeling of this process. The results of this endeavor, representing the most significant part of our contribution to the problem under discussion, are presented in Section 2.2.

Our initial intention was to faithfully reanalyze the same data set as was used in [1].  However, it became clear that the Novartis Gene Atlas data set is not amenable to correlation analysis because it represents a mix of arrays derived from diverse biological specimens, each being of a different origin and each representing a single copy of the corresponding set of expression measurements. In other words, these data do not represent a random sample, defined as a sequence of independent and identically distributed random vectors, which is required for a statistically sound inference on correlation coefficients. If one chooses to ignore this fact and produces sample correlation coefficients from such data, the resultant estimates will not be interpretable in probabilistic terms and their statistical properties, such as consistency, will be uncertain. Therefore, the histograms of pairwise correlation coefficients presented in [1] are statistically invalid. It is an observation drawn from the same (homogeneous) general population that adds information on an unknown parameter, and not an observation generated from a dissimilar distribution. The Novartis Gene Atlas and like data sets would have been amenable to statistical analysis had they included multiple independent replicates of each tissue type. Without this important feature, any inferences from such data are not generalizable to the general population and make little sense both statistically and biologically. Unfortunately, this aspect of the problem is frequently neglected in the bioinformatics literature. The mixture-based approach discussed by Dr. Gaile in his review does not circumvent the obstacle because the Novartis Gene Atlas typically provides only a single observation per each component of the underlying mixture of distributions. In response to Dr. Jordan's comment, we can only express our regret at the fact that statistical principles are violated too often in this field of research.

The second problem has to do with data normalization. We have discussed adverse effects of normalization in conjunction with single-color microarrays in several publications [2,5-7]. Leaving aside the question of whether or not the currently used normalization procedures achieve their promulgated goal, it is a well-known fact that they distort to various degrees the correlation structure of microarray data [5,8], the latter being the main concern in reference to the results reported in [1]. The popular view that the observed correlations between gene expression levels are solely attributable to an array-specific random effect caused by the technical noise is demonstrably false [2,9,10]. Recall that, in accordance with our analysis of the MAQC data set, the level of random fluctuations of gene expression signals attributable to the technical noise in the contemporary Affymetrix platform is too low to cause a tangible bias in estimated correlation coefficients [2]. There is also independent evidence that normalization procedures distort the joint distribution of the true expression signals quite dramatically, even affecting their marginal distributions [6]. Every known normalization procedure resorts to pooling (heavily dependent) observed signals across different probes (probe sets), thereby producing surrogate variables whose distributions differ from those of the true biological signals. In the context of testing for differential expression, this distortion of the true signal may induce an uncontrollable number of false discoveries, an effect especially pronounced in large sample studies where control of type 1 errors may be entirely lost. The adverse effects of normalization procedures will be addressed more comprehensively in a forthcoming paper. We find it beyond reason to resort to normalization when assessing the effects of cross-hybridization on the correlation structure of microarray gene expression data.

We respectfully disagree with Dr. Jordan that normalized data can be of some utility in correlation analysis. The popular belief that normalization should be universally applied to microarray data has already caused a great deal of harm to numerous biological and methodological studies. The idea of normalization was initially offered as an *ad hoc *expedient to improve significance testing for differentially expressed genes in two-sample comparisons. Even in this setting, the universal benefits of normalization are questionable (see above). The situation is more obvious when the main focus is on correlation coefficients. Destroying correlations before studying them quantitatively does not make any sense to us. This is exactly the pitfall biologists should be aware of in order to avoid false biological conclusions. Therefore, we maintain the opinion that normalization should not be used when making inferences about the correlation structure of microarray data.

For the reasons presented above, we turned to two other data sets that meet the requirements of correlation analysis. The choice of data sets was governed by the quest for larger sample sizes and the need for the same array type (HG_U133A) as was used in [1]. Our analysis of the chosen data sets appeared to be in conflict with the observations reported in [1]. Yet another conflict became apparent when comparing our stochastic description of cross-hybridization (Section 2.2.1) with the simulation model employed by Okoniewski and Miller.

Unlike Ploner et al. [8], we see no reason to swiftly accept the conjecture that the majority of gene pairs must be composed of independent genes, while trying to explain the actually observed strong and long-range correlations in the majority of gene pairs [9-12] by "non-biological" causes. (The term long-range correlation refers to the situation where a given gene displays large correlation coefficients, say, greater than 0.8, with thousands of other genes). In view of our study of the random component of technical noise [2], we find it much more plausible that the observed positive correlations reflect the true biological correlations, whether it be a manifestation of transcriptional regulation or confounding due to heterogeneity of cell populations [13,14]. There is some additional evidence to support this opinion. In particular, such evidence is provided by the phenomenon of type A dependence described in [9]. This mass phenomenon manifests itself beyond a very conservative estimate of the level of technical noise. Our updated estimate suggests that the proportion of type A pairs with very high positive correlation coefficients is close to 50% [10]. In accordance with a mathematical argument given in [10], the abundance of this type of stochastic dependence leads us to conclude that positive true correlations dominate the correlation structure of microarray data. Yet another argument in the context of cross-hybridization will be given in Section 2.

The term "cross-hybridization" refers to a complex physical/chemical process and is not entirely reducible to the concept of "multiple targeting" in its simplistic form amenable to probabilistic modeling. In the context of this paper, however, the two terms will be used interchangeably. The present paper attempts to illuminate the following two questions:

(1) Can the process of cross-hybridization lead to strong positive correlations in families of those probe sets that are known to have the potential for multiple targeting?

(2) If the answer to the previous question is "yes", is this effect prevalent enough to manifest itself in a tangible proportion of gene pairs so that, e.g., the average (over all gene pairs) is affected?

The authors of [1] gave a positive answer to both questions. In view of the results reported in the present paper, we are inclined to a positive answer to the first question and to a negative answer to the second one. Furthermore, we see no serious evidence to support concerns about the utility of estimated correlation coefficients in microarray data analysis. The understanding of cross-hybridization yielded by our study is much more complex than Question 1 implies. There is a diversity of effects that are theoretically possible, including a substantial increase in the strength of positive correlation. Therefore, we do not dispute the statement by Okoniewski and Miller that MT can alter the estimates of correlation coefficients constructed from microarray data, albeit the problem is definitely not as extreme as presented by these authors (see Section 2.1 for details). It is also worth noting that the capacity of cross-hybridization to induce negative correlations has been overlooked in previous publications.

The results of data analysis and an alternative theoretical model are presented and discussed at length in the next section.

## 2. Results and discussion

### 2.1. Experimentation with biological data

We begin by testing the results reported in [1]. We used the following two data sets for this purpose:

(1) A set of data on breast cancer cells cultured *in vitro *[15]. Only "vehicle" control samples treated with the medium used to solubilize the inhibitor were included in our analysis. The sample size equals *n *= 48 in this study. In what follows we will refer to this collection of data as Data Set 1.

(2) A subset of the data published in [16]. This subset refers to *n *= 61 untreated patients with breast cancer. RNA samples were obtained at the John Radcliffe Hospital (Oxford, UK) and processed at the Jules Bordet Institute in Brussels, Belgium. Patients with all grades and ER status were included in the sample under study. In this set of microarray data, referred to as Data Set 2, correlations between gene expression levels tend to be weaker than in Data Set 1, as well as in all other data sets we have interrogated so far [10]. We selected Data Set 2 as a sort of extreme example to make our case stronger.

Both studies were carried out with HG_U133A Affymetrix arrays for which Okoniewski and Miller [1] identified 3859 non-overlapping families containing MT probe sets with an average number of 2.56 probe sets per family. The total number of MT probe sets is 9875. Each such probe set contains off-target reporters suggested by sequence analysis. For simplicity, we will refer to all MT probe sets as "bad" probe sets. By contrast, the probe sets other than those included in the families will be referred to as "good" probe sets. For the same reason, the terms "probe set" and "gene" will be used interchangeably. A total of 12340 probe sets are not considered problematic (bad) on the grounds of sequence analysis.

**Figure 1:**
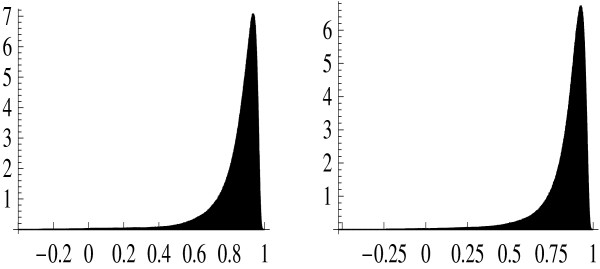
Histogram of correlation coefficients for pairs of good (A) and bad (B) probe sets. Data Set 1.

**Figure 2:**
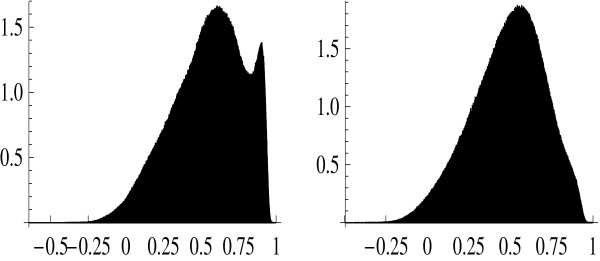
Histogram of correlation coefficients for pairs of good (A) and bad (B) probe sets. Data Set 2.

Since the required computations for all (i.e., ~ 2.5 × 10^8^) probe set pairs are quite time-consuming, a subset of size 5000 was randomly drawn (without replacement) from the set of bad probe sets. The Pearson correlation coefficients were computed for all pairs of genes contained in this subset. A subset of the same size was randomly drawn (without replacement) from the set of good probe sets, yielding the pair-wise correlation coefficients in the same manner. Figure 1 presents the histogram of correlation coefficients for good and bad probe sets in Data Set 1. This histogram is intended to show the abundance of gene pairs with different correlation coefficients; it does not have any statistical meaning, of course. No tangible difference between bad and good probe sets is seen when comparing the two histograms shown in Figure 1. This is obvious from their numerical characteristics such as mean and standard deviation. The mean (across genes) value of correlation coefficient equals 0.841 for bad probe sets while it is equal to 0.847 for good probe sets. The corresponding standard deviations are equal to 0.133 and 0.132, respectively. The situation is similar in Data Set 2 (Figure 2) with a more pronounced tendency for bad probe sets to have even smaller correlation coefficients than their good counterparts. In this case, the mean values are 0.488 for bad probe sets and 0.550 for good ones, while the respective standard deviations are 0.216 and 0.238. The irregular shape of the histogram in Figure 2A (reflecting heterogeneity of this particular data set) does not affect our conclusion because both the mean value and the variance tend to be smaller for bad probe sets.

The above-described experimentation with microarray data does not prove that the effects of cross-hybridization are non-existent in the Affymetrix GeneChip platform. On the contrary, the process of multiple targeting is expected to affect the true correlation between genes in accordance with the theoretical considerations presented in Section 2.2. A more practical question, however, is whether such effects are strong and abundant enough to change the estimates of correlations coefficients in a large subset of genes. That the process of cross-hybridization restricted to the families of MT probe sets cannot be considered a mass phenomenon follows from the mere fact that the abundance of bad pairs is low. Indeed, the total number of all gene pairs is of order 10^8^. Given the pairs of bad probe sets overlap only within each family, the total number of such pairs is of order 10^4^, a very small portion in the ocean of all pairs. This shows that the results of our comparison of "bad" versus "good" probe sets are reasonably expected.

While the above experimentation does not support the conclusion by Okoniewski and Miller [1] drawn from huge numbers of correlation coefficients, it is worth considering more local effects restricted to individual families of MT probe sets. This is exactly the point made by Dr. Jordan in his review. In many instances, we were unable to detect any perceptible effect of cross-hybridization in terms of correlation coefficients. A typical example is presented in Table 1. Shown in Table 1 is the correlation matrix for all pairs of probe sets from two problematic families, each consisting of two MT probe sets. These estimates indicate little dissimilarity between the intra-family and inter-family pairs. Unfortunately, no formal statistical test can be applied to compare the estimated correlation coefficients because the expression levels in the pairs of probe sets under consideration are stochastically dependent. Nonetheless, this example demonstrates that the potential for cross-hybridization may remain untapped even if the presence of some off-target reporters is suggested by the analysis of transcript sequences.

**Table 1:** Correlation coefficients in all pairs of probe sets from two problematic families. Probe sets 1 and 2 pertain to the first family while probe sets 3 and 4 to the second. Since the correlation matrix is symmetric, only the elements above its diagonal are presented. The within-family elements are given in italics. Data Set 1.

Probe sets	1	2	3	4
1	-	*0.955*	0962	0.942
2	-	-	0.946	0.927
3	-	-	-	*0.983*
4	-	-	-	-

While our analysis does not support concerns about adverse effects of cross-hybridization on the correlation structure of microarray data, this cannot be considered a conclusive proof because a certain degree of non-specific binding for good probe sets cannot be completely ruled out. Estimating the contribution of this conceivable effect to the correlations observed in microarray data is a difficult and probably impracticable task because the processes of cross-hybridization cannot be observed directly. Using theoretical modeling, however, some simpler questions seem approachable. They are loosely formulated as follows:

1. What effects of cross-hybridization on the correlation structure of microarray data are theoretically possible? What are the probabilistic characteristics of transcripts targeted by multiple probe sets that drive such effects?

2. Can the observed long-range correlation structure of microarray data be attributed solely to non-specific binding of multiple ("bad" and "good" alike) probe sets to a putative common transcript, assuming that this transcript, if it exists, is highly abundant and capable of affecting the correlations between expression measures even if its affinity to probe sets is low?

The utility of model-based inference on cross-hybridization effects is considered in the next section.

### 2.2. Theoretical considerations

#### 2.2.1. The case of two competing probe sets

In this section, we consider the simplest case of two probe sets targeting the same transcript. Much like the microarray technology in general, our theory will be based on the following assumption:

*Assumption 1*. No saturation effects are present and the true signal intensity (disregarding the technical measurement error) is proportional to the amount of RNA bound to a given probe set with a constant (non-random) coefficient of proportionality. The coefficients for different probe sets do not have to be the same.

*Remark 1*. We tested Assumption 1 using the data set on various spiked-in probes (GeneChip HG_U133A) provided by the file HG_U133A_tag_Latin_Square.zip on the Affymetrix website. For some spiked-in probes, the dependence of the fluorescence intensity on the molar concentration was indeed linear, but deviations from linearity were also noted. The small sample size (*n *= 3) in this study and its sub-optimal design that uses a non-linear scale for the independent variable still leave room for speculations. A more rigorous metrological study is required to validate Assumption 1 governing the overall usefulness of modern high-density oligonucleotide microarray technology.

Proceeding from Assumption 1, suppose that two probe sets compete for the same (common) transcript so that the first probe set binds to this transcript with probability *p*, and, given the transcript remains unbound, the second probe set binds to it with probability 1 - *p*. Denote the amount of the common transcripts (RNA molecules) by *ν*. The model considered by Okoniewski and Miller [1] assumes that the signal intensities *Z*_1 _and *Z*_2 _for the two probe sets can be represented as *Z*_1 _= *pν*, *Z*_2 _= (1 - *p*)*ν*, which is why its simulation counterpart always displays a positive covariance. In mechanistic terms, these relationships imply that each probe set "knows" exactly what proportion of the random amount *ν *it must "catch" in the course of hybridization, thereby making this essentially deterministic model highly implausible.

A more natural model can be constructed if one proceeds from a stochastic nature of cross-hybridization and represents the first probe set signal as

Z1=∑j=1νξj,
 MathType@MTEF@5@5@+=feaafiart1ev1aaatCvAUfKttLearuWrP9MDH5MBPbIqV92AaeXatLxBI9gBaebbnrfifHhDYfgasaacPC6xNi=xI8qiVKYPFjYdHaVhbbf9v8qqaqFr0xc9vqFj0dXdbba91qpepeI8k8fiI+fsY=rqGqVepae9pg0db9vqaiVgFr0xfr=xfr=xc9adbaqaaeGacaGaaiaabeqaaeqabiWaaaGcbaGaemOwaO1aaSbaaSqaaiabigdaXaqabaGccqGH9aqpdaaeWbqaaGGaciab=57a4naaBaaaleaacqWGQbGAaeqaaaqaaiabdQgaQjabg2da9iabigdaXaqaaiab=17aUbqdcqGHris5aOGaeiilaWcaaa@3B04@

where *ξ*_*j *_are independent and identically distributed (i.i.d.) indicator variables taking on values 1 and 0 with the probabilities *p *and 1 - *p*, respectively. For the second probe set we have

Z2=∑j=1ν(1−ξj).
 MathType@MTEF@5@5@+=feaafiart1ev1aaatCvAUfKttLearuWrP9MDH5MBPbIqV92AaeXatLxBI9gBaebbnrfifHhDYfgasaacPC6xNi=xI8qiVKYPFjYdHaVhbbf9v8qqaqFr0xc9vqFj0dXdbba91qpepeI8k8fiI+fsY=rqGqVepae9pg0db9vqaiVgFr0xfr=xfr=xc9adbaqaaeGacaGaaiaabeqaaeqabiWaaaGcbaGaemOwaO1aaSbaaSqaaiabikdaYaqabaGccqGH9aqpdaaeWbqaaiabcIcaOiabigdaXiabgkHiTGGaciab=57a4naaBaaaleaacqWGQbGAaeqaaOGaeiykaKIaeiOla4caleaacqWGQbGAcqGH9aqpcqaIXaqmaeaacqWF9oGBa0GaeyyeIuoaaaa@3EA4@

The mutually dependent random variables (r.v.s) *Z*_1 _and *Z*_2 _satisfy the condition: *Z*_1 _+ *Z*_2 _= *ν*. It is easy to verify that the covariance between *Z*_1 _and *Z*_2 _can be of any sign under this model. Indeed,

Cov(Z1,Z2)=E{∑j=1νξj∑k=1ν(1−ξk)}−E{∑j=1νξj}E{∑k=1ν(1−ξk)}=p(1−p)E{ν(ν−1)}−p(1−p)(E{ν})2=p(1−p)(Var{ν}−E{ν}),
 MathType@MTEF@5@5@+=feaafiart1ev1aaatCvAUfKttLearuWrP9MDH5MBPbIqV92AaeXatLxBI9gBaebbnrfifHhDYfgasaacPC6xNi=xI8qiVKYPFjYdHaVhbbf9v8qqaqFr0xc9vqFj0dXdbba91qpepeI8k8fiI+fsY=rqGqVepae9pg0db9vqaiVgFr0xfr=xfr=xc9adbaqaaeGacaGaaiaabeqaaeqabiWaaaGcbaqbaeqabiqaaaqaaiabboeadjabb+gaVjabbAha2jabcIcaOiabdQfaAnaaBaaaleaacqaIXaqmaeqaaOGaeiilaWIaemOwaO1aaSbaaSqaaiabikdaYaqabaGccqGGPaqkcqGH9aqptuuDJXwAK1uy0HMmaeHbfv3ySLgzG0uy0HgiuD3BaGabaiab=ri8fjabcUha7naaqahabaacciGae4NVdG3aaSbaaSqaaiabdQgaQbqabaaabaGaemOAaOMaeyypa0JaeGymaedabaGae4xVd4ganiabggHiLdGcdaaeWbqaaiabcIcaOiabigdaXiabgkHiTiab+57a4naaBaaaleaacqWGRbWAaeqaaOGaeiykaKIaeiyFa0haleaacqWGRbWAcqGH9aqpcqaIXaqmaeaacqGF9oGBa0GaeyyeIuoakiabgkHiTiab=ri8fjabcUha7naaqahabaGae4NVdG3aaSbaaSqaaiabdQgaQbqabaGccqGG9bqFaSqaaiabdQgaQjabg2da9iabigdaXaqaaiab+17aUbqdcqGHris5aOGae8hHWxKaei4EaS3aaabCaeaacqGGOaakcqaIXaqmcqGHsislcqGF+oaEdaWgaaWcbaGaem4AaSgabeaakiabcMcaPiabc2ha9bWcbaGaem4AaSMaeyypa0JaeGymaedabaGae4xVd4ganiabggHiLdGccqGH9aqpaeaacqWGWbaCcqGGOaakcqaIXaqmcqGHsislcqWGWbaCcqGGPaqkcqWFecFrcqGG7bWEcqGF9oGBcqGGOaakcqGF9oGBcqGHsislcqaIXaqmcqGGPaqkcqGG9bqFcqGHsislcqWGWbaCcqGGOaakcqaIXaqmcqGHsislcqWGWbaCcqGGPaqkcqGGOaakcqWFecFrcqGG7bWEcqGF9oGBcqGG9bqFcqGGPaqkdaahaaWcbeqaaiabikdaYaaakiabg2da9iabdchaWjabcIcaOiabigdaXiabgkHiTiabdchaWjabcMcaPiabcIcaOiabbAfawjabbggaHjabbkhaYjabcUha7jab+17aUjabc2ha9jabgkHiTiab=ri8fjabcUha7jab+17aUjabc2ha9jabcMcaPiabcYcaSaaaaaa@C37D@

so that the sign of Cov (*Z*_1_, *Z*_2_) depends on the relationship between Var{*ν*} and E
 MathType@MTEF@5@5@+=feaafiart1ev1aaatCvAUfKttLearuWrP9MDH5MBPbIqV92AaeXatLxBI9gBaebbnrfifHhDYfgasaacPC6xNi=xH8viVGI8Gi=hEeeu0xXdbba9frFj0xb9qqpG0dXdb9aspeI8k8fiI+fsY=rqGqVepae9pg0db9vqaiVgFr0xfr=xfr=xc9adbaqaaeGacaGaaiaabeqaaeqabiWaaaGcbaWefv3ySLgznfgDOjdaryqr1ngBPrginfgDObcv39gaiqaacqWFecFraaa@37B3@{*ν*}, both parameters being unobservable, of course. In particular, Cov (*Z*_1_, *Z*_2_) = 0 if *ν *has a Poisson distribution. We find it very interesting that the sign of correlation is entirely determined by probabilistic characteristics of the common transcript and not by its affinity to the competing probe sets, the latter being quantified by the probability *p*.

We agree with Dr. Gaile that one can easily derive the correlation coefficient between *Z*_1 _and *Z*_2 _by the same straightforward argument. However, we resort to another derivation based on the notion of probability generating function (p.g.f.), which is instructive to obtain the needed results in Section 2.2.2. Introduce the p.g.f. of the random variable (r.v.) *ν*:

Pν(u)=Euν.
 MathType@MTEF@5@5@+=feaafiart1ev1aaatCvAUfKttLearuWrP9MDH5MBPbIqV92AaeXatLxBI9gBaebbnrfifHhDYfgasaacPC6xNi=xI8qiVKYPFjYdHaVhbbf9v8qqaqFr0xc9vqFj0dXdbba91qpepeI8k8fiI+fsY=rqGqVepae9pg0db9vqaiVgFr0xfr=xfr=xc9adbaqaaeGacaGaaiaabeqaaeqabiWaaaGcbaGaeeiuaa1aaSbaaSqaaGGaciab=17aUbqabaGccqGGOaakcqWG1bqDcqGGPaqkcqGH9aqptuuDJXwAK1uy0HMmaeHbfv3ySLgzG0uy0HgiuD3BaGabaiab+ri8fjabdwha1naaCaaaleqabaGae8xVd4gaaOGaeiOla4caaa@4388@

Then the Laplace transform *f*(*s, t*) of the joint distribution of the vector (*Z*_1_, *Z*_2_) assumes the form

f(s,t)=E{e−sZ1−tZ2}=E{e−s∑j=1νξj−t∑j=1ν(1−ξj)}=Pν(pe−s+(1−p)e−t).
 MathType@MTEF@5@5@+=feaafiart1ev1aaatCvAUfKttLearuWrP9MDH5MBPbIqV92AaeXatLxBI9gBaebbnrfifHhDYfgasaacPC6xNi=xI8qiVKYPFjYdHaVhbbf9v8qqaqFr0xc9vqFj0dXdbba91qpepeI8k8fiI+fsY=rqGqVepae9pg0db9vqaiVgFr0xfr=xfr=xc9adbaqaaeGacaGaaiaabeqaaeqabiWaaaGcbaGaemOzayMaeiikaGIaem4CamNaeiilaWIaemiDaqNaeiykaKIaeyypa0Zefv3ySLgznfgDOjdaryqr1ngBPrginfgDObcv39gaiqaacqWFecFrcqGG7bWEcqWGLbqzdaahaaWcbeqaaiabgkHiTiabdohaZjabdQfaAnaaBaaameaacqaIXaqmaeqaaSGaeyOeI0IaemiDaqNaemOwaO1aaSbaaWqaaiabikdaYaqabaaaaOGaeiyFa0Naeyypa0Jae8hHWxKaei4EaSNaemyzau2aaWbaaSqabeaacqGHsislcqWGZbWCdaaeWaqaaGGaciab+57a4naaBaaameaacqWGQbGAaeqaaSGaeyOeI0IaemiDaqhameaacqWGQbGAcqGH9aqpcqaIXaqmaeaacqGF9oGBa4GaeyyeIuoalmaaqadabaGaeiikaGIaeGymaeJaeyOeI0Iae4NVdG3aaSbaaWqaaiabdQgaQbqabaWccqGGPaqkaWqaaiabdQgaQjabg2da9iabigdaXaqaaiab+17aUbGdcqGHris5aaaakiabc2ha9jabg2da9iabbcfaqnaaBaaaleaacqGF9oGBaeqaaOGaeiikaGIaemiCaaNaemyzau2aaWbaaSqabeaacqGHsislcqWGZbWCaaGccqGHRaWkcqGGOaakcqaIXaqmcqGHsislcqWGWbaCcqGGPaqkcqWGLbqzdaahaaWcbeqaaiabgkHiTiabdsha0baakiabcMcaPiabc6caUaaa@8873@

Differentiating log *f*(*s, t*) twice (with respect to both *s *and *t*), one can derive the covariance between *Z*_1 _and *Z*_2_. The variance of *Z*_1 _can be obtained by differentiating log *f*(*s, t*) twice with respect to *s*. In like manner, one obtains the variance of *Z*_2 _by differentiating log *f*(*s, t*) twice with respect to *t*. This leads to the following formula for the correlation coefficient between *Z*_1 _and *Z*_2_:



Introducing the notation *κ *= Var{*ν*}/E
 MathType@MTEF@5@5@+=feaafiart1ev1aaatCvAUfKttLearuWrP9MDH5MBPbIqV92AaeXatLxBI9gBaebbnrfifHhDYfgasaacPC6xNi=xH8viVGI8Gi=hEeeu0xXdbba9frFj0xb9qqpG0dXdb9aspeI8k8fiI+fsY=rqGqVepae9pg0db9vqaiVgFr0xfr=xfr=xc9adbaqaaeGacaGaaiaabeqaaeqabiWaaaGcbaWefv3ySLgznfgDOjdaryqr1ngBPrginfgDObcv39gaiqaacqWFecFraaa@37B3@{*ν*} and taking into account that

P′ν(1)=Eν,P″ν(1)=Eν2−Eν,
 MathType@MTEF@5@5@+=feaafiart1ev1aaatCvAUfKttLearuWrP9MDH5MBPbIqV92AaeXatLxBI9gBaebbnrfifHhDYfgasaacPC6xNi=xI8qiVKYPFjYdHaVhbbf9v8qqaqFr0xc9vqFj0dXdbba91qpepeI8k8fiI+fsY=rqGqVepae9pg0db9vqaiVgFr0xfr=xfr=xc9adbaqaaeGacaGaaiaabeqaaeqabiWaaaGcbaqbaeqabeGaaaqaaiqbbcfaqzaafaWaaSbaaSqaaGGaciab=17aUbqabaGccqGGOaakcqaIXaqmcqGGPaqkcqGH9aqptuuDJXwAK1uy0HMmaeHbfv3ySLgzG0uy0HgiuD3BaGabaiab+ri8fjab=17aUjabcYcaSaqaaiqbbcfaqzaagaWaaSbaaSqaaiab=17aUbqabaGccqGGOaakcqaIXaqmcqGGPaqkcqGH9aqpcqGFecFrcqWF9oGBdaahaaWcbeqaaiabikdaYaaakiabgkHiTiab+ri8fjab=17aUjabcYcaSaaaaaa@530B@

formula (3) assumes the form:

Corr(Z1,Z2)=p(1−p)(κ−1)p2κ+p(1−p)(1−p)2κ+p(1−p).
 MathType@MTEF@5@5@+=feaafiart1ev1aaatCvAUfKttLearuWrP9MDH5MBPbIqV92AaeXatLxBI9gBaebbnrfifHhDYfgasaacPC6xNi=xI8qiVKYPFjYdHaVhbbf9v8qqaqFr0xc9vqFj0dXdbba91qpepeI8k8fiI+fsY=rqGqVepae9pg0db9vqaiVgFr0xfr=xfr=xc9adbaqaaeGacaGaaiaabeqaaeqabiWaaaGcbaacbaGae83qamKae83Ba8Mae8NCaiNae8NCaiNaeiikaGIaemOwaO1aaSbaaSqaaiabigdaXaqabaGccqGGSaalcqWGAbGwdaWgaaWcbaGaeGOmaidabeaakiabcMcaPiabg2da9KqbaoaalaaabaGaemiCaaNaeiikaGIaeGymaeJaeyOeI0IaemiCaaNaeiykaKIaeiikaGccciGae4NUdSMaeyOeI0IaeGymaeJaeiykaKcabaWaaOaaaeaacqWGWbaCdaahaaqabeaacqaIYaGmaaGae4NUdSMaey4kaSIaemiCaaNaeiikaGIaeGymaeJaeyOeI0IaemiCaaNaeiykaKcabeaadaGcaaqaaiabcIcaOiabigdaXiabgkHiTiabdchaWjabcMcaPmaaCaaabeqaaiabikdaYaaacqGF6oWAcqGHRaWkcqWGWbaCcqGGOaakcqaIXaqmcqGHsislcqWGWbaCcqGGPaqkaeqaaaaakiabc6caUaaa@6185@

**Figure 3:**
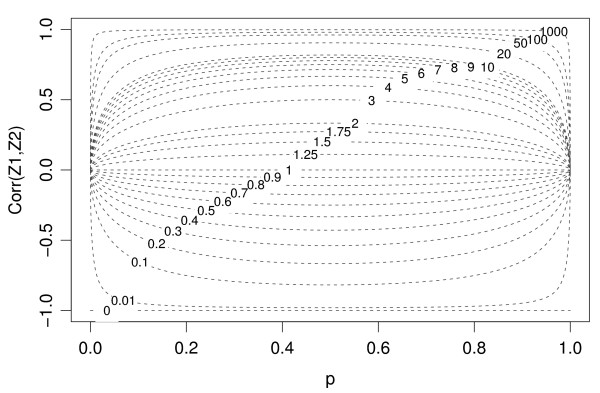
The behavior of Corr(*Z*_1_, *Z*_2_)as a function of *p *for different values of the parameter *k*. This figure was provided by Dr. Gaile in his review.

Figure 3 shows the behavior of Corr(*Z*_1_, *Z*_2_) as a function of *p *for different values of the parameter *k*. We thank Dr. Gaile for this figure that illustrates the parametric family given by formula (4). The right-hand side of (4) attains a maximum at *p *= 0.5 and consequently

κ≥1+Corr(Z1,Z2)1−Corr(Z1,Z2).
 MathType@MTEF@5@5@+=feaafiart1ev1aaatCvAUfKttLearuWrP9MDH5MBPbIqV92AaeXatLxBI9gBaebbnrfifHhDYfgasaacPC6xNi=xI8qiVKYPFjYdHaVhbbf9v8qqaqFr0xc9vqFj0dXdbba91qpepeI8k8fiI+fsY=rqGqVepae9pg0db9vqaiVgFr0xfr=xfr=xc9adbaqaaeGacaGaaiaabeqaaeqabiWaaaGcbaacciGae8NUdSMaeyyzImBcfa4aaSaaaeaacqaIXaqmcqGHRaWkieaacqGFdbWqcqGFVbWBcqGFYbGCcqGFYbGCcqGGOaakcqWGAbGwdaWgaaqaaiabigdaXaqabaGaeiilaWIaemOwaO1aaSbaaeaacqaIYaGmaeqaaiabcMcaPaqaaiabigdaXiabgkHiTiab+neadjab+9gaVjab+jhaYjab+jhaYjabcIcaOiabdQfaAnaaBaaabaGaeGymaedabeaacqGGSaalcqWGAbGwdaWgaaqaaiabikdaYaqabaGaeiykaKcaaOGaeiOla4caaa@4DBC@

For example, if the model is valid for a particular pair (*Z*_1_, *Z*_2_) and Corr(*Z*_1_, *Z*_2_) ≥ 0.9, then it follows from inequality (5) that Var{*ν*} ≥ 19 E
 MathType@MTEF@5@5@+=feaafiart1ev1aaatCvAUfKttLearuWrP9MDH5MBPbIqV92AaeXatLxBI9gBaebbnrfifHhDYfgasaacPC6xNi=xH8viVGI8Gi=hEeeu0xXdbba9frFj0xb9qqpG0dXdb9aspeI8k8fiI+fsY=rqGqVepae9pg0db9vqaiVgFr0xfr=xfr=xc9adbaqaaeGacaGaaiaabeqaaeqabiWaaaGcbaWefv3ySLgznfgDOjdaryqr1ngBPrginfgDObcv39gaiqaacqWFecFraaa@37B3@{*ν*}. In Data Set 1, 53.6% of bad probe sets and 39.4% of good probe sets satisfy this condition. However, this does not mean that the process of cross-hybridization plays a substantial role in the correlation structure of microarray data for the following reasons:

1. Inequality (5) provides a necessary but not a sufficient condition for the correlation Corr(*Z*_1_, *Z*_2_) to exceed a certain level.

2. Condition (5) applies only to those isolated gene pairs where the total amount of the target transcript is eventually bound to one of the two probe sets and no other transcripts can contribute to their ultimate expression measures (recall Section 2.1).

While the above model, designed for a single pair of probe sets competing for the same transcript, is instructive to study general regularities in the process of cross-hybridization, it is obviously of limited utility in studying probable causes of the observed long-range correlation that extends over thousands of genes. A more general model of the non-specific binding process for arbitrary pairs formed from multiple probe sets is considered in the next section.

#### 2.2.2. Multiple probe sets

In Section 2.2.1, we confirmed that the process of cross-hybridization can induce spurious positive correlations within certain families of probe sets whose potential for multiple targeting is suggested by the analysis of transcript sequences. At the same time, our analysis of Section 2.1 shows that the overall correlation structure of gene expression data cannot be driven by the minority of non-overlapping pairs of such problematic probe sets. The analysis of transcript sequences and homologies, however, is only suggestive and we have to consider the case where the event of non-specific binding is admissible for virtually all probe sets, even if its probability for each of them is low. Presented below is a simplistic model that attempts to describe the process of massive cross-hybridization involving all probe sets.

Suppose there exists a large pool of common RNA molecules that have the ability to bind to any of the *k *probe sets under consideration. Under the proposed model, each probe set binds first to its own highly specific transcript without any competition with other probe sets (i.e., with probability 1) and then it binds to a portion of the common transcript in the competitive fashion. More specifically, each molecule of the common transcript is bound to the *j*th probe set with probability *p*_*j *_≥ 0, *j *= 1,...,*k*, and the probabilities *p*_*j *_satisfy the condition: ∑j=1kpj=1
 MathType@MTEF@5@5@+=feaafiart1ev1aaatCvAUfKttLearuWrP9MDH5MBPbIqV92AaeXatLxBI9gBaebbnrfifHhDYfgasaacPC6xNi=xH8viVGI8Gi=hEeeu0xXdbba9frFj0xb9qqpG0dXdb9aspeI8k8fiI+fsY=rqGqVepae9pg0db9vqaiVgFr0xfr=xfr=xc9adbaqaaeGacaGaaiaabeqaaeqabiWaaaGcbaWaaabmaeaacqWGWbaCdaWgaaWcbaGaemOAaOgabeaakiabg2da9iabigdaXaWcbaGaemOAaOMaeyypa0JaeGymaedabaGaem4AaSganiabggHiLdaaaa@3779@

Denote the amount of a specific transcript bound to the *j*th probe set with probability 1 by *X*_*j*_. To make our model identifiable, we need additionally the following basic assumption: 

*Assumption 2*. The r.v.s *X*_*j*_, *j *= 1,...,*k*, are mutually independent.

Assumption 2 is not intended for a realistic description of gene expression signals but rather as a reference for measuring cross-hybridization effects. Recall that Question 2 posed at the end of Section 2.1 explicitly refers to this assumption. Therefore, the results that follow should be interpreted under a hypothetical scenario attributing all of the observable correlation to the competition between multiple probe sets for the above-postulated common transcript.

Assuming the same mechanism of competition for the common transcript, and invoking the same independence assumptions as in Section 2.2.1, we write

Zj=Xj+∑l=1νεlj,j=1,...,k,
 MathType@MTEF@5@5@+=feaafiart1ev1aaatCvAUfKttLearuWrP9MDH5MBPbIqV92AaeXatLxBI9gBaebbnrfifHhDYfgasaacPC6xNi=xI8qiVKYPFjYdHaVhbbf9v8qqaqFr0xc9vqFj0dXdbba91qpepeI8k8fiI+fsY=rqGqVepae9pg0db9vqaiVgFr0xfr=xfr=xc9adbaqaaeGacaGaaiaabeqaaeqabiWaaaGcbaqbaeqabeGaaaqaaiabdQfaAnaaBaaaleaacqWGQbGAaeqaaOGaeyypa0JaemiwaG1aaSbaaSqaaiabdQgaQbqabaGccqGHRaWkdaaeWbqaaGGaciab=v7aLnaaDaaaleaacqWGSbaBaeaacqWGQbGAaaaabaGaemiBaWMaeyypa0JaeGymaedabaGae8xVd4ganiabggHiLdGccqGGSaalaeaacqWGQbGAcqGH9aqpcqaIXaqmcqGGSaalcqGGUaGlcqGGUaGlcqGGUaGlcqGGSaalcqWGRbWAcqGGSaalaaaaaa@4A74@

where *Z*_*j *_is the total RNA amount bound to the *j*th probe set, εlj
 MathType@MTEF@5@5@+=feaafiart1ev1aaatCvAUfKttLearuWrP9MDH5MBPbIqV92AaeXatLxBI9gBaebbnrfifHhDYfgasaacPC6xNi=xH8viVGI8Gi=hEeeu0xXdbba9frFj0xb9qqpG0dXdb9aspeI8k8fiI+fsY=rqGqVepae9pg0db9vqaiVgFr0xfr=xfr=xc9adbaqaaeGacaGaaiaabeqaaeqabiWaaaGcbaacciGae8xTdu2aa0baaSqaaiabdYgaSbqaaiabdQgaQbaaaaa@306C@ are indicators taking on a value of 1 with probability *p*_*j *_and of 0 with probability 1 - *p*_*j*_, ∑j=1kεlj=1
 MathType@MTEF@5@5@+=feaafiart1ev1aaatCvAUfKttLearuWrP9MDH5MBPbIqV92AaeXatLxBI9gBaebbnrfifHhDYfgasaacPC6xNi=xH8viVGI8Gi=hEeeu0xXdbba9frFj0xb9qqpG0dXdb9aspeI8k8fiI+fsY=rqGqVepae9pg0db9vqaiVgFr0xfr=xfr=xc9adbaqaaeGacaGaaiaabeqaaeqabiWaaaGcbaWaaabmaeaaiiGacqWF1oqzdaqhaaWcbaGaemiBaWgabaGaemOAaOgaaaqaaiabdQgaQjabg2da9iabigdaXaqaaiabdUgaRbqdcqGHris5aOGaeyypa0JaeGymaedaaa@3915@, and the random vectors (εl11,...,εl1k)
 MathType@MTEF@5@5@+=feaafiart1ev1aaatCvAUfKttLearuWrP9MDH5MBPbIqV92AaeXatLxBI9gBaebbnrfifHhDYfgasaacPC6xNi=xH8viVGI8Gi=hEeeu0xXdbba9frFj0xb9qqpG0dXdb9aspeI8k8fiI+fsY=rqGqVepae9pg0db9vqaiVgFr0xfr=xfr=xc9adbaqaaeGacaGaaiaabeqaaeqabiWaaaGcbaGaeiikaGccciGae8xTdu2aa0baaSqaaiabdYgaSnaaBaaameaacqaIXaqmaeqaaaWcbaGaeGymaedaaOGaeiilaWIaeiOla4IaeiOla4IaeiOla4IaeiilaWIae8xTdu2aa0baaSqaaiabdYgaSnaaBaaameaacqaIXaqmaeqaaaWcbaGaem4AaSgaaOGaeiykaKcaaa@3D10@ and (εl21,...,εl2k)
 MathType@MTEF@5@5@+=feaafiart1ev1aaatCvAUfKttLearuWrP9MDH5MBPbIqV92AaeXatLxBI9gBaebbnrfifHhDYfgasaacPC6xNi=xH8viVGI8Gi=hEeeu0xXdbba9frFj0xb9qqpG0dXdb9aspeI8k8fiI+fsY=rqGqVepae9pg0db9vqaiVgFr0xfr=xfr=xc9adbaqaaeGacaGaaiaabeqaaeqabiWaaaGcbaGaeiikaGccciGae8xTdu2aa0baaSqaaiabdYgaSnaaBaaameaacqaIYaGmaeqaaaWcbaGaeGymaedaaOGaeiilaWIaeiOla4IaeiOla4IaeiOla4IaeiilaWIae8xTdu2aa0baaSqaaiabdYgaSnaaBaaameaacqaIYaGmaeqaaaWcbaGaem4AaSgaaOGaeiykaKcaaa@3D14@ are independent for *l*_1 _≠ *l*_2_. The r.v. *ν *represents the total number of molecules of the common transcript available for the competition between different probe sets. To convert the quantity *Z*_*j *_into the corresponding expression intensity in accordance with Assumption 1, one needs to account for the fact that the specific and common transcripts may vary in size (mass). Without any loss of generality, this can easily be accomplished if the unit of measurement of the variables *X*_*j *_is taken to be the molecular weight of the common transcript.

Recalling the definition of the p.g.f. P
 MathType@MTEF@5@5@+=feaafiart1ev1aaatCvAUfKttLearuWrP9MDH5MBPbIqV92AaeXatLxBI9gBaebbnrfifHhDYfgasaacPC6xNi=xH8viVGI8Gi=hEeeu0xXdbba9frFj0xb9qqpG0dXdb9aspeI8k8fiI+fsY=rqGqVepae9pg0db9vqaiVgFr0xfr=xfr=xc9adbaqaaeGacaGaaiaabeqaaeqabiWaaaGcbaGaeeiuaafaaa@2CFA@ (Section 2.2.1), the joint characteristic function *g *of the random vector *Z*_1 _,...,*Z*_*k *_can be represented as

g(t1,...,tk)=∏j=1kgj(tj)P(∑j=1kpjeitj),
 MathType@MTEF@5@5@+=feaafiart1ev1aaatCvAUfKttLearuWrP9MDH5MBPbIqV92AaeXatLxBI9gBaebbnrfifHhDYfgasaacPC6xNi=xI8qiVKYPFjYdHaVhbbf9v8qqaqFr0xc9vqFj0dXdbba91qpepeI8k8fiI+fsY=rqGqVepae9pg0db9vqaiVgFr0xfr=xfr=xc9adbaqaaeGacaGaaiaabeqaaeqabiWaaaGcbaGaem4zaCMaeiikaGIaemiDaq3aaSbaaSqaaiabigdaXaqabaGccqGGSaalcqGGUaGlcqGGUaGlcqGGUaGlcqGGSaalcqWG0baDdaWgaaWcbaGaem4AaSgabeaakiabcMcaPiabg2da9maarahabaGaem4zaC2aaSbaaSqaaiabdQgaQbqabaGccqGGOaakcqWG0baDdaWgaaWcbaGaemOAaOgabeaakiabcMcaPaWcbaGaemOAaOMaeyypa0JaeGymaedabaGaem4AaSganiabg+GivdGccqqGqbaucqGGOaakdaaeWbqaaiabdchaWnaaBaaaleaacqWGQbGAaeqaaOGaemyzau2aaWbaaSqabeaacqWGPbqAcqWG0baDdaWgaaadbaGaemOAaOgabeaaaaGccqGGPaqkaSqaaiabdQgaQjabg2da9iabigdaXaqaaiabdUgaRbqdcqGHris5aOGaeiilaWcaaa@5C59@

where *g*_*j *_is the marginal characteristic function of *X*_*j*_, *j *= 1,...,*k*. The covariance between two r.v.s equals a minus mixed second order log-derivative of their characteristic function evaluated at zero. From expression (7), we have

Cov(Zr,Zs)=(σν2−Eν)prps
 MathType@MTEF@5@5@+=feaafiart1ev1aaatCvAUfKttLearuWrP9MDH5MBPbIqV92AaeXatLxBI9gBaebbnrfifHhDYfgasaacPC6xNi=xI8qiVKYPFjYdHaVhbbf9v8qqaqFr0xc9vqFj0dXdbba91qpepeI8k8fiI+fsY=rqGqVepae9pg0db9vqaiVgFr0xfr=xfr=xc9adbaqaaeGacaGaaiaabeqaaeqabiWaaaGcbaGaee4qamKaee4Ba8MaeeODayNaeiikaGIaemOwaO1aaSbaaSqaaiabdkhaYbqabaGccqGGSaalcqWGAbGwdaWgaaWcbaGaem4CamhabeaakiabcMcaPiabg2da9iabcIcaOGGaciab=n8aZnaaDaaaleaacqWF9oGBaeaacqaIYaGmaaGccqGHsisltuuDJXwAK1uy0HMmaeHbfv3ySLgzG0uy0HgiuD3BaGabaiab+ri8fjab=17aUjabcMcaPiabdchaWnaaBaaaleaacqWGYbGCaeqaaOGaemiCaa3aaSbaaSqaaiabdohaZbqabaaaaa@5447@

for *r *= 1,...,*k*, *s *= 1,...,*k*, *r *≠ = *s*. Here σν2
 MathType@MTEF@5@5@+=feaafiart1ev1aaatCvAUfKttLearuWrP9MDH5MBPbIqV92AaeXatLxBI9gBaebbnrfifHhDYfgasaacPC6xNi=xH8viVGI8Gi=hEeeu0xXdbba9frFj0xb9qqpG0dXdb9aspeI8k8fiI+fsY=rqGqVepae9pg0db9vqaiVgFr0xfr=xfr=xc9adbaqaaeGacaGaaiaabeqaaeqabiWaaaGcbaacciGae83Wdm3aa0baaSqaaiab=17aUbqaaiabikdaYaaaaaa@306F@ is the variance of the r.v. *ν*. Using the condition

∑r=1k∑s=1kprps=1,
 MathType@MTEF@5@5@+=feaafiart1ev1aaatCvAUfKttLearuWrP9MDH5MBPbIqV92AaeXatLxBI9gBaebbnrfifHhDYfgasaacPC6xNi=xI8qiVKYPFjYdHaVhbbf9v8qqaqFr0xc9vqFj0dXdbba91qpepeI8k8fiI+fsY=rqGqVepae9pg0db9vqaiVgFr0xfr=xfr=xc9adbaqaaeGacaGaaiaabeqaaeqabiWaaaGcbaWaaabCaeaadaaeWbqaaiabdchaWnaaBaaaleaacqWGYbGCaeqaaOGaemiCaa3aaSbaaSqaaiabdohaZbqabaGccqGH9aqpcqaIXaqmaSqaaiabdohaZjabg2da9iabigdaXaqaaiabdUgaRbqdcqGHris5aaWcbaGaemOCaiNaeyypa0JaeGymaedabaGaem4AaSganiabggHiLdGccqGGSaalaaa@4325@

from (8) we obtain the formula

(σν2−Eν)(1−∑j=1kpj2)=∑r≠sCov(Zr,Zs).
 MathType@MTEF@5@5@+=feaafiart1ev1aaatCvAUfKttLearuWrP9MDH5MBPbIqV92AaeXatLxBI9gBaebbnrfifHhDYfgasaacPC6xNi=xI8qiVKYPFjYdHaVhbbf9v8qqaqFr0xc9vqFj0dXdbba91qpepeI8k8fiI+fsY=rqGqVepae9pg0db9vqaiVgFr0xfr=xfr=xc9adbaqaaeGacaGaaiaabeqaaeqabiWaaaGcbaGaeiikaGccciGae83Wdm3aa0baaSqaaiab=17aUbqaaiabikdaYaaakiabgkHiTmrr1ngBPrwtHrhAYaqeguuDJXwAKbstHrhAGq1DVbaceaGae4hHWxKae8xVd4MaeiykaKIaeiikaGIaeGymaeJaeyOeI0YaaabCaeaacqWGWbaCdaqhaaWcbaGaemOAaOgabaGaeGOmaidaaaqaaiabdQgaQjabg2da9iabigdaXaqaaiabdUgaRbqdcqGHris5aOGaeiykaKIaeyypa0ZaaabuaeaacqqGdbWqcqqGVbWBcqqG2bGDcqGGOaakcqWGAbGwdaWgaaWcbaGaemOCaihabeaakiabcYcaSiabdQfaAnaaBaaaleaacqWGZbWCaeqaaOGaeiykaKcaleaacqWGYbGCcqGHGjsUcqWGZbWCaeqaniabggHiLdGccqGGUaGlaaa@6452@

Note that the right-hand side of formula (9) can be estimated from observed expression measures. Minimizing ∑j=1kpj2
 MathType@MTEF@5@5@+=feaafiart1ev1aaatCvAUfKttLearuWrP9MDH5MBPbIqV92AaeXatLxBI9gBaebbnrfifHhDYfgasaacPC6xNi=xH8viVGI8Gi=hEeeu0xXdbba9frFj0xb9qqpG0dXdb9aspeI8k8fiI+fsY=rqGqVepae9pg0db9vqaiVgFr0xfr=xfr=xc9adbaqaaeGacaGaaiaabeqaaeqabiWaaaGcbaWaaabmaeaacqWGWbaCdaqhaaWcbaGaemOAaOgabaGaeGOmaidaaaqaaiabdQgaQjabg2da9iabigdaXaqaaiabdUgaRbqdcqGHris5aaaa@3661@ with respect to *p*_*j *_under the constraint ∑j=1kpj=1
 MathType@MTEF@5@5@+=feaafiart1ev1aaatCvAUfKttLearuWrP9MDH5MBPbIqV92AaeXatLxBI9gBaebbnrfifHhDYfgasaacPC6xNi=xH8viVGI8Gi=hEeeu0xXdbba9frFj0xb9qqpG0dXdb9aspeI8k8fiI+fsY=rqGqVepae9pg0db9vqaiVgFr0xfr=xfr=xc9adbaqaaeGacaGaaiaabeqaaeqabiWaaaGcbaWaaabmaeaacqWGWbaCdaWgaaWcbaGaemOAaOgabeaakiabg2da9iabigdaXaWcbaGaemOAaOMaeyypa0JaeGymaedabaGaem4AaSganiabggHiLdaaaa@3779@, we have

σν2−Eν≥kk−1∑r≠sCov(Zr,Zs)≥∑r≠sCov(Zr,Zs)=γ.
 MathType@MTEF@5@5@+=feaafiart1ev1aaatCvAUfKttLearuWrP9MDH5MBPbIqV92AaeXatLxBI9gBaebbnrfifHhDYfgasaacPC6xNi=xI8qiVKYPFjYdHaVhbbf9v8qqaqFr0xc9vqFj0dXdbba91qpepeI8k8fiI+fsY=rqGqVepae9pg0db9vqaiVgFr0xfr=xfr=xc9adbaqaaeGacaGaaiaabeqaaeqabiWaaaGcbaacciGae83Wdm3aa0baaSqaaiab=17aUbqaaiabikdaYaaakiabgkHiTmrr1ngBPrwtHrhAYaqeguuDJXwAKbstHrhAGq1DVbaceaGae4hHWxKae8xVd4MaeyyzImBcfa4aaSaaaeaacqWGRbWAaeaacqWGRbWAcqGHsislcqaIXaqmaaWaaabuaeaacqqGdbWqcqqGVbWBcqqG2bGDaeaacqWGYbGCcqGHGjsUcqWGZbWCaeqacqGHris5aiabcIcaOiabdQfaAnaaBaaabaGaemOCaihabeaacqGGSaalcqWGAbGwdaWgaaqaaiabdohaZbqabaGaeiykaKIaeyyzIm7aaabuaeaacqqGdbWqcqqGVbWBcqqG2bGDcqGGOaakcqWGAbGwdaWgaaqaaiabdkhaYbqabaGaeiilaWIaemOwaO1aaSbaaeaacqWGZbWCaeqaaiabcMcaPaqaaiabdkhaYjabgcMi5kabdohaZbqabiabggHiLdGaeyypa0Jae83SdCMaeiOla4caaa@7111@

The latter inequality allows us to estimate a lower bound for σν2−Eν
 MathType@MTEF@5@5@+=feaafiart1ev1aaatCvAUfKttLearuWrP9MDH5MBPbIqV92AaeXatLxBI9gBaebbnrfifHhDYfgasaacPC6xNi=xH8viVGI8Gi=hEeeu0xXdbba9frFj0xb9qqpG0dXdb9aspeI8k8fiI+fsY=rqGqVepae9pg0db9vqaiVgFr0xfr=xfr=xc9adbaqaaeGacaGaaiaabeqaaeqabiWaaaGcbaacciGae83Wdm3aa0baaSqaaiab=17aUbqaaiabikdaYaaakiabgkHiTmrr1ngBPrwtHrhAYaqeguuDJXwAKbstHrhAGq1DVbaceaGae4hHWxKae8xVd4gaaa@3EF8@.

To use inequality (10), one needs to compute covariances for all gene pairs. For the GeneChip HG_U133A, this means conducting such computations for all pairs formed from 12340 genes, which is computationally prohibitive. For this reason, we used the St. Jude Hospital Children's Research Hospital Database produced with U95 arrays [17]. Specifically, we used a subset of data that reports expression levels of *k *= 7084 genes in *n *= 79 patients with childhood leukemia (TELL type). In this data set, probe sets with dubious definitions [18] were removed using the custom CDF file from [19], which gives reason for us to believe that our analysis refers predominantly to "good" probe sets. The estimated value of the lower bound *γ *in inequality (10) for these probe sets is γ^
 MathType@MTEF@5@5@+=feaafiart1ev1aaatCvAUfKttLearuWrP9MDH5MBPbIqV92AaeXatLxBI9gBaebbnrfifHhDYfgasaacPC6xNi=xH8viVGI8Gi=hEeeu0xXdbba9frFj0xb9qqpG0dXdb9aspeI8k8fiI+fsY=rqGqVepae9pg0db9vqaiVgFr0xfr=xfr=xc9adbaqaaeGacaGaaiaabeqaaeqabiWaaaGcbaacciGaf83SdCMbaKaaaaa@2D91@ = 8.74 × 10^11^.

**Figure 4:**
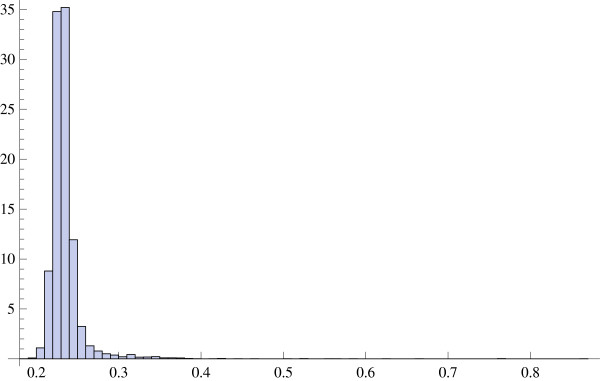
Variation coefficients for gene expression levels in the TELL data set.

There are two ways of interpreting the estimate γ^
 MathType@MTEF@5@5@+=feaafiart1ev1aaatCvAUfKttLearuWrP9MDH5MBPbIqV92AaeXatLxBI9gBaebbnrfifHhDYfgasaacPC6xNi=xH8viVGI8Gi=hEeeu0xXdbba9frFj0xb9qqpG0dXdb9aspeI8k8fiI+fsY=rqGqVepae9pg0db9vqaiVgFr0xfr=xfr=xc9adbaqaaeGacaGaaiaabeqaaeqabiWaaaGcbaacciGaf83SdCMbaKaaaaa@2D91@ resulted from our analysis. First, we proceed from the obvious inequality: σν2>8.74×1011
 MathType@MTEF@5@5@+=feaafiart1ev1aaatCvAUfKttLearuWrP9MDH5MBPbIqV92AaeXatLxBI9gBaebbnrfifHhDYfgasaacPC6xNi=xH8viVGI8Gi=hEeeu0xXdbba9frFj0xb9qqpG0dXdb9aspeI8k8fiI+fsY=rqGqVepae9pg0db9vqaiVgFr0xfr=xfr=xc9adbaqaaeGacaGaaiaabeqaaeqabiWaaaGcbaacciGae83Wdm3aa0baaSqaaiab=17aUbqaaiabikdaYaaakiabg6da+iabiIda4iabc6caUiabiEda3iabisda0iabgEna0kabigdaXiabicdaWmaaCaaaleqabaGaeGymaeJaeGymaedaaaaa@3B57@. The average (across genes) variance of gene expression levels in this data set is 3.5 × 10^4^, which is at least seven orders of magnitude lower than that of the common transcript given by the above inequality. This huge variance of *ν *seems quite implausible if the common transcript is thought of as being produced by one of the typical protein-encoding genes such as those that are measured by the standard microarray technology. Second, we can gain some insight into the magnitude of the mean value of *ν*. Introducing the notation *μ *= E
 MathType@MTEF@5@5@+=feaafiart1ev1aaatCvAUfKttLearuWrP9MDH5MBPbIqV92AaeXatLxBI9gBaebbnrfifHhDYfgasaacPC6xNi=xH8viVGI8Gi=hEeeu0xXdbba9frFj0xb9qqpG0dXdb9aspeI8k8fiI+fsY=rqGqVepae9pg0db9vqaiVgFr0xfr=xfr=xc9adbaqaaeGacaGaaiaabeqaaeqabiWaaaGcbaWefv3ySLgznfgDOjdaryqr1ngBPrginfgDObcv39gaiqaacqWFecFraaa@37B3@*ν*, we represent inequality (11) in the form

vν2μ2−μ≥kk−1∑r≠sCov(Zr,Zs)≥∑r≠sCov(Zr,Zs),
 MathType@MTEF@5@5@+=feaafiart1ev1aaatCvAUfKttLearuWrP9MDH5MBPbIqV92AaeXatLxBI9gBaebbnrfifHhDYfgasaacPC6xNi=xI8qiVKYPFjYdHaVhbbf9v8qqaqFr0xc9vqFj0dXdbba91qpepeI8k8fiI+fsY=rqGqVepae9pg0db9vqaiVgFr0xfr=xfr=xc9adbaqaaeGacaGaaiaabeqaaeqabiWaaaGcbaGaemODay3aa0baaSqaaGGaciab=17aUbqaaiabikdaYaaakiab=X7aTnaaCaaaleqabaGaeGOmaidaaOGaeyOeI0Iae8hVd0MaeyyzImBcfa4aaSaaaeaacqWGRbWAaeaacqWGRbWAcqGHsislcqaIXaqmaaWaaabuaeaacqqGdbWqcqqGVbWBcqqG2bGDcqGGOaakcqWGAbGwdaWgaaqaaiabdkhaYbqabaGaeiilaWIaemOwaO1aaSbaaeaacqWGZbWCaeqaaiabcMcaPiabgwMiZoaaqafabaGaee4qamKaee4Ba8MaeeODayNaeiikaGIaemOwaO1aaSbaaeaacqWGYbGCaeqaaiabcYcaSiabdQfaAnaaBaaabaGaem4CamhabeaacqGGPaqkaeaacqWGYbGCcqGHGjsUcqWGZbWCaeqacqGHris5aaqaaiabdkhaYjabgcMi5kabdohaZbqabiabggHiLdGaeiilaWcaaa@6515@

where *v *= *σ*_*ν*_/*μ *is the variation coefficient of the r.v. *ν*. Figure 4 displays the histogram of variation coefficients in the TELL data. The histogram is extremely narrow, indicating that the variation coefficient is effectively constant across genes. The same fact was documented by Wu and Irizarry [20]. In the TELL data set, the mean variation coefficient is equal to 0.235. Using this value as an estimate of *v *and solving the quadratic inequality (11) with respect to *μ*, we have *μ *> 3.98 × 10^6^. This lower bound for *μ *is quite close to the mean total expression, i.e., the sum of the mean values of all gene expressions, the latter being equal to 4.22 × 10^6^.

The unrealistically huge mean and variance of the hypothetical common transcript targeted by multiple probe sets make it very unlikely that a massive cross-hybridization manifests itself at the probe set level. Put another way, if such a putative transcript exists and induces the observed long-range correlation between gene expression signals by binding to multiple probe sets, it cannot be a typical protein-encoding transcript, irrespective of whether its affinity to the probe sets is high or low. Nor can the random technical noise cause this kind of correlation between gene expression levels as follows from our analysis of the MAQC data [2]. This leads us to conclude that the observed correlation structure of microarray data is of a biological nature. There are several conceivable biological causes of correlations between gene expression signals such as regulatory processes engaged in gene function, composite cellular make-up of tissues, and latent heterogeneity of subjects. We see no way to estimate their contributions from microarray data because of identifiability problems.

**Figure 5:**
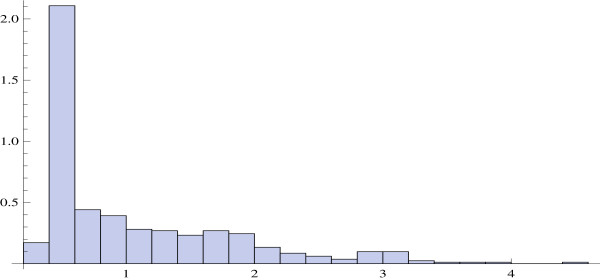
Variation coefficients for expression levels of miRNAs in SKBr3 breast cancer cells.

*Remark 2*. Regulatory interactions between genes participating in biochemical pathways or networks may (or may not) cause only a short-range correlation or the so-called clumpy dependence [21]. Superimposed on this causal dependence are the effects caused by different species of noncoding RNA implicated in regulation of large sets of genes. The global term "noncoding RNA" (ncRNA) refers to a large class of transcripts that do not encode a protein product. An important subclass of functional ncRNAs is represented by microRNAs (miRNAs). These small, typically 21-25nt long, transcripts have been subject of intense studies in recent years. Using either miRNA transfection into cultured cells [22] or miRNA antagonists *in vivo *[23], it has been shown that a particular miRNA may affect hundreds of genes by interfering with their transcripts. This is a large-scale effect but it is still doubtful whether the observed long-range correlation between gene expression levels, involving thousands of genes, can be exhaustively explained by this mechanism. The two major modes of miRNA action are mRNA cleavage and translational inhibition. In the latter case, all untranslated mRNAs are eventually fated to degradation as well. While there is some similarity between such effects and those of cross-hybridization (binding to a common transcript), they call for a different stochastic model that would allow for the cognate mRNA degradation. Nevertheless, it is interesting to see whether the expression levels of miRNA are subject to a much higher variation than those mRNAs presented in Figure 4. Figure 5 displays variation coefficients of expression levels for different miRNA in SKBr3 breast cancer cells (untreated controls, *n *= 38) produced by spotted oligonucleotide microarrays. The data were retrieved from the Gene Expression Omnibus Database (see [24], GSE3798). It is clear that some classes of miRNA are much more variable than any of the protein-coding mRNAs in Figure 4, despite the fact that the inter-sample variability is generally expected to be lower *in vitro *than *in vivo*. This suggests that, when trying to explain the nature of long-range correlations in gene expression data involving gigantic sets of genes, one should look more closely at the "dark matter" of ncRNAs and confounding effects caused by heterogeneity of biological tissues or/and subjects [13,14,25] rather than at technical flaws of microarray technology.

The above characteristics of the putative common transcript were obtained from a set of 7084 genes. It is conceivable that numerous genes outside of this set may also contribute to the effect of cross-hybridization even if they are not physically present on the array. The question arises as to whether our estimates can be extrapolated to the whole totality of genes. There is no definitive answer to this question. On the one hand, if the covariances between gene expression levels are expected to be predominantly positive, the right hand sides of inequalities (11) and (12) may only increase when additional pairs are formed from the complementary genes. In this special sense, the estimates given by inequalities (10) and (11) are conservative. It is also important that they are independent of specific values of *p*_*j*_. On the other hand, it cannot be entirely ruled out that a heretofore unknown gene (probe set) with the above-described peculiar features or a formidable number of negatively correlated gene pairs will emerge from future data sets. Therefore, the result reported here is intermediate by nature as it relies on the present-day knowledge and plausibility arguments.

## 3. Conclusion

It is now a well-known fact that numerous probe sets have the potential for multiple targeting in accordance with the results of sequence analysis. While it is theoretically possible that multiple targeting may lead to both negative and positive correlations between expression signals in gene pairs, the observed correlations in families of such problematic probe sets are typically positive and strong. However, they are also overwhelmingly positive and strong in pairs of those probe sets with a high specificity to the corresponding unique transcripts. Our experimentation with microarray data produced by high-density oligonucleotide microarray technology leads us to conclude that the observed long-range positive correlation in the majority of gene pairs cannot be attributed to the presence of problematic probe sets with the potential for cross-hybridization. Some aspects of the problem of cross-hybridization are aproachable by means of probabilistic modeling despite the fact that this process is not directly observable in microarray data.

## 4. Methods

The statistical methods employed in this paper are intertwined closely with the reported results and cannot be presented in a separate section. All the necessary technical details of data analysis and data sources are given Section 2.

## Reviewers' comments

### Review 1 (I.K. Jordan)

Klebanov and colleagues revisit the effect of oligonucleotide microarray design on the inference of correlation coefficients used to detect coexpression between genes. The authors of this study had previously concluded that the level of technical noise in the widely used Affymetrix oligonucleotide microarray platform was low and thus did not present any major obstacle for the analysis and interpretation of experimental results (Klebanov and Yakolev Biology Direct 2007 2: 9). A corollary of this finding was the fact that the effects of cross-hybridization between probes and targets would be random and insubstantial. However, a recently published paper reached a very different conclusion. Okoniewski and Miller (BMC Bioinformatics 2006 7: 276) published an analysis of widely used microarray datasets and conclude that oligonucleotide microarrays are prone to false positives correlations due to multiple targeting. If this were to be the case, it would have implications for hundreds of studies. Accordingly, this issue is both timely and highly relevant.

Klebanov et al. dispute the findings of Okoniewski and Miller based on the appropriateness of the dataset analyzed and the adequacy of the simulation model used. Since I am not a statistician, I am not qualified to evaluate the nuances of the simulation models proposed. I will confine my comments and questions to the more biological aspects of the manuscript. The analysis presented here is quite sound and convincing in a strict sense. For the data analyzed by Klebanov et al., there is no systematic bias in correlation coefficient calculations introduced by multiple targeting. However, in a more general sense, it seems that the some of the differences between the two studies Klebanov et al. versus Okoniewski and Miller -have to do with different methodological approaches that are necessitated by statistical versus biological questions. Of course, this is not to say that statistics and biology are mutually exclusive; clearly, sound biological conclusions require robust statistical inferences. Nevertheless, choices about the structure of data sets to be analyzed can be quite different for a statistician versus a biologist. This epistemological reality should be addressed by Klebanov et al. before dismissing out-of-hand the relevance of the results of Okoniewski and Miller. I go into more detail on this issue below.

1. It would help to set the stage for the results that are presented if what is meant by the "process of multiple targeting in short oligonucleotide microarrays" is precisely defined at the outset of the introduction. For instance, the relationship between the terms multiple targeting and cross-hybridization could be clarified for the naive reader.

2. Klebanov et al. point out two problems with the Novartis Gene Atlas dataset and analysis used by Okoniewski and Miller to assess the effects of multiple targeting on correlation analysis. The first problem is that this dataset does not represent a random sample as defined in statistical terms, i.e. "a sequence of independent and identically distributed random vectors, which is required for statistically sound inference of correlation coefficients." Accordingly, it will not be possible to statistically (probabilistically) interpret correlation coefficients computed from such data. This may be statistically undeniable, but the particular structure of the Novartis Gene Atlas, "a mix of arrays from diverse biological specimens", is exactly what makes it interesting and relevant from a biological perspective. Biologists will be most interested in learning which genes are coexpressed across similar sets of tissues in such a dataset, and in fact the two Novartis Gene Atlas papers have been cited over 800 times. Therefore, the statistical properties of inferences made on such data will be biologically germane.

3. The second problem the authors have with the analysis of Okoniewski and Miller has to do with the normalization procedure used. They cite several reports, which indicate that normalization distorts the correlations obtained from microarray datasets. They go on to state unequivocally that "We find it beyond reason to resort to normalization when assessing the effects of cross-hybridization on the correlation structure of microarray expression data." While this may indeed be the case from a statistical perspective, the fact is that the vast majority of microarray analyses do employ a normalization step prior to the calculation of correlation coefficients. This may be statistically problematic for the reasons the authors point out but it represents the modus operandi of microarray studies. Therefore, for a biologist who is interested in understanding potential pitfalls in the analysis of his or her data, it may not be unreasonable at all to investigate the correlation properties of normalized data.

4. The good versus bad probe-set correlation coefficient histograms do indeed look indistinguishable in Figure 1 as the authors claim but the 'good' histogram in Figure 2 looks different with a distinct second peak as r approaches a value of 1. This is only alluded to as a higher average r for the good set. Can the authors further explain and clarify this trend for the reader?

5. The point about the relatively small size of bad probe-set pairs, ~ 10^4 ^versus ~ 10^8 ^total pairs, is not entirely convincing. This relatively small size may mean that the overall statistical properties of the inferred correlations are not substantially affected as the authors point out. However, if the observations that are of particular interest to the biologist are included in this set then the confounding effects of cross-hybridization could still be relevant. 6. Is the computation of correlation coefficients really so time consuming as to require the use of a random subset of 5,000 probes instead of the analysis of all the data? Analysis of the entire data set, or at least a bootstrapping type of procedure, would be more convincing.

The e-mail exchange between I. K. Jordan and A. Yakovlev that followed this review is given below:

**I. K. Jordan**: Attached please find my review of your paper. As you will see, I find your statistical results entirely convincing. However, I think there needs to be some acknowledgement and discussion of the relevance of different datasets and analytical approaches of biologists versus statisticians. A discussion of this kind will strengthen your paper and make your conclusions more relevant to biologists. I go into detail on this matter in my review.

**A. Yakovlev**: Thank you very much. Your review is very thoughtful as usual. We disagree on only one issue. In my opinion, no inference based on invalid statistical inference can be biologically meaningful. I will try to address your concerns to the extent possible for a statistician.

**I. K. Jordan**: Your point is very well taken. It can only be true, as you say, that "no inference based on invalid statistical inference can be biologically meaningful." I think you will find little (reasonable) disagreement on that issue. The point I was trying to make may not be quite so straightforward though. Biologists may well be interested in the behavior, or properties, of sets of correlation coefficients drawn from sets of experiments that, when considered as an ensemble, do not make up a true set of random variables. While it may be difficult (impossible?) to evaluate the behavior of such a set of observations in a formal probabilistic framework, it may still be relevant, or at least useful, to consider how the correlations behave with respect to properties of the experimental system such as 'good' versus 'bad' sets of probes. Thus, the use of formal models to capture the behavior may not be justified, but simple observations could still provide some insight. Does that make any more sense? None of this in any way invalidates your own analysis or model. It only provides some common ground for statisticians and biologists to appreciate their different, but not mutually exclusive, perspectives.

**A. Yakovlev**: While biologists and statisticians are equally interested in the truth, they have different perceptions of the scientific rigor as far as data analysis is concerned. Consider the following real story. Suppose a paper published some 10 years ago suggests that all microarray data be normalized by dividing the expression level of each gene on a given array by the arithmetic mean taken over all genes on the same array. Sounds familiar, is it not? The author claims (without any theoretical proof) that this will remove the systematic bias and increase the power of two-sample tests. At first glance, it looks like a reasonable way to go. If you are a biologist, you will or will not ask: why? Most likely, you will just follow the advice out of your respect to mathematics. After a short time, this procedure becomes a dogma and you have to apply it routinely even if you have begun experiencing serious reservations based on your practical experience. You know that refraining from data normalization may bring you trouble with your reviewers. If you are a statistician, you must ask where this claim follows from and how it can be underpinned by a theoretical argument or at least by simulations. If simulations are provided, you must question their realism and look for possible side effects that may devalue the benefits, if any, of the proposed procedure. If the procedure is beneficial only under a certain condition, a natural question would be how to test the presence of such a condition with real data, because, in the absence of a reliable test, the procedure may still be too risky to apply. For example, the above-mentioned normalization procedure replaces one bias with another, which may or may not have serious consequences depending on other factors such as the effect and sample sizes. This procedure also reduces correlations in gene pairs, which may sometimes be beneficial in two-sample settings but much less so where the main focus is on correlations. If the procedure has been tested using artificial spiked-in probes, you should ask whether this is a legitimate test from the statistical standpoint (by the way, it is not). I can go on and on, but even this simple example demonstrates very well that biologists put too much of their trust in *ad hoc *methods that have no or little theoretical justification. They should have been much tougher on their "methodological" colleagues, requiring that the proposed statistical (or other analytical) method be substantiated and tested much more rigorously. Biologists need to know what the proposed methodologies do and what they do not do. In my opinion, a formidable amount of false biological knowledge has been accumulated over the years and this is the most serious problem faced by modern bioinformatics and computational biology.

Other responses are provided in the text.

### Review 2 (D. P. Gaile)

This manuscript addresses open (and important) research questions regarding the effects of cross hybridization (CH) on gene by gene correlation estimates. The authors argue that the effects of multiple targeting on correlation based inference may not be as serious/prevalent as is suggested in a recent paper by Okoniewski and Miller. To that end and beyond, two models are developed to promote a better understanding the effects of cross hybridization. The first model is developed to address questions related to multiple targeting and the second model is developed to address the hypothesis that all probe sets may compete (to varying extents) for a common transcript. Data from two gene expression array datasets are used to obtain empirical estimates for the unknown model parameters and several conclusions are drawn. These conclusions are well reasoned and justified provided one is willing to accept the clearly stated assumptions. Unfortunately, the adequacy of the proposed models can not be directly evaluated due to limitations in current technologies and experimental practices and so the results of these analyses, while of value, are admittedly speculative.

1. Klebanov et al. claim that the data used by Okoniewski and Miller are not amenable to correlation analysis. This criticism is warranted as Okoniewski and Miller failed to identify and properly characterize which samples were used in their analyses. Although the sample array data were generated as part of the Gene Atlas project, Okoniewski and Miller do not identify exactly which samples were used. Their simulation studies were based on a subset of 50 arrays yet, surprisingly, no details are provided regarding the identity and method of selection of said array data. Perhaps one could think of the data as an approximately random sample on a mixture population. Namely, one could consider the transcript quantities for the *I *samples *ν*_1_, ..., *ν*_1 _to be (approximately) a random sample from fv(v)=∑k=1Kπkgk(v)
 MathType@MTEF@5@5@+=feaafiart1ev1aaatCvAUfKttLearuWrP9MDH5MBPbIqV92AaeXatLxBI9gBaebbnrfifHhDYfgasaacPC6xNi=xH8viVGI8Gi=hEeeu0xXdbba9frFj0xb9qqpG0dXdb9aspeI8k8fiI+fsY=rqGqVepae9pg0db9vqaiVgFr0xfr=xfr=xc9adbaqaaeGacaGaaiaabeqaaeqabiWaaaGcbaGaemOzay2aaSbaaSqaaiabdAha2bqabaGccqGGOaakcqWG2bGDcqGGPaqkcqGH9aqpdaaeWaqaaGGaciab=b8aWnaaBaaaleaacqWGRbWAaeqaaOGaem4zaC2aaSbaaSqaaiabdUgaRbqabaGccqGGOaakcqWG2bGDcqGGPaqkaSqaaiabdUgaRjabg2da9iabigdaXaqaaiabdUealbqdcqGHris5aaaa@42E2@, where *g*_*k*_(*ν*) represents the distribution of transcript counts for the *k*th tumor population. Under this model,

Var(ν)E(ν)=∑kπk(σk2+μk2)−(∑kπkμk)2∑kπkμk,
 MathType@MTEF@5@5@+=feaafiart1ev1aaatCvAUfKttLearuWrP9MDH5MBPbIqV92AaeXatLxBI9gBaebbnrfifHhDYfgasaacPC6xNi=xI8qiVKYPFjYdHaVhbbf9v8qqaqFr0xc9vqFj0dXdbba91qpepeI8k8fiI+fsY=rqGqVepae9pg0db9vqaiVgFr0xfr=xfr=xc9adbaqaaeGacaGaaiaabeqaaeqabiWaaaGcbaqcfa4aaSaaaeaacqqGwbGvcqqGHbqycqqGYbGCcqGGOaakiiGacqWF9oGBcqGGPaqkaeaatuuDJXwAK1uy0HMmaeHbfv3ySLgzG0uy0HgiuD3BaGabaiab+ri8fjabcIcaOiab=17aUjabcMcaPaaacqGH9aqpdaWcaaqaamaaqafabaGae8hWda3aaSbaaeaacqWGRbWAaeqaaiabcIcaOiab=n8aZnaaDaaabaGaem4AaSgabaGaeGOmaidaaiabgUcaRiab=X7aTnaaDaaabaGaem4AaSgabaGaeGOmaidaaiabcMcaPiabgkHiTmaabmaabaWaaabuaeaacqWFapaCdaWgaaqaaiabdUgaRbqabaGae8hVd02aaSbaaeaacqWGRbWAaeqaaaqaaiabdUgaRbqabiabggHiLdaacaGLOaGaayzkaaWaaWbaaeqabaGaeGOmaidaaaqaaiabdUgaRbqabiabggHiLdaabaWaaabuaeaacqWFapaCdaWgaaqaaiabdUgaRbqabaGae8hVd02aaSbaaeaacqWGRbWAaeqaaaqaaiabdUgaRbqabiabggHiLdaaaiabcYcaSaaa@6E0C@

which can provide values that are larger than max⁡kσk2μk
 MathType@MTEF@5@5@+=feaafiart1ev1aaatCvAUfKttLearuWrP9MDH5MBPbIqV92AaeXatLxBI9gBaebbnrfifHhDYfgasaacPC6xNi=xH8viVGI8Gi=hEeeu0xXdbba9frFj0xb9qqpG0dXdb9aspeI8k8fiI+fsY=rqGqVepae9pg0db9vqaiVgFr0xfr=xfr=xc9adbaqaaeGacaGaaiaabeqaaeqabiWaaaGcbaWaaCbeaeaacyGGTbqBcqGGHbqycqGG4baEaSqaaiabdUgaRbqabaqcfa4aaSaaaeaaiiGacqWFdpWCdaqhaaqaaiabdUgaRbqaaiabikdaYaaaaeaacqWF8oqBdaWgaaqaaiabdUgaRbqabaaaaaaa@399D@. [Note: σk2
 MathType@MTEF@5@5@+=feaafiart1ev1aaatCvAUfKttLearuWrP9MDH5MBPbIqV92AaeXatLxBI9gBaebbnrfifHhDYfgasaacPC6xNi=xH8viVGI8Gi=hEeeu0xXdbba9frFj0xb9qqpG0dXdb9aspeI8k8fiI+fsY=rqGqVepae9pg0db9vqaiVgFr0xfr=xfr=xc9adbaqaaeGacaGaaiaabeqaaeqabiWaaaGcbaacciGae83Wdm3aa0baaSqaaiabdUgaRbqaaiabikdaYaaaaaa@301B@, *μ*_*k *_correspond to the variance and mean, respectively, of a random variable with distribution *g*_*k*_(*ν*)]. This highlights the fact that the heterogeneous collection of samples can lead to inflated values of the variation coefficient and hence can produce pronounced (and possibly atypical) CH effects on correlation estimates. So, even if one is willing to accept that the correlation estimates provided by Okoniewski and Miller have meaning, than there is still the issue of whether or not the magnitude and nature of the CH effects observed in that data set should be considered typical.

2. The inclusion of a figure to illustrate equation 4 would be helpful. An example is provided (Figure 3). The plotted lines are labeled according to the kappa value used to generate them. 3. The use of conditional expectations provides a much clearer (and shorter) derivation of equation (4). For example, E(Z1Z2)Eν[E(Z1(ν−Z1)|ν]=Eν[ν2p−νp(1−p)+ν2p2]=p(1−p)(Eνν2−Eνν)
 MathType@MTEF@5@5@+=feaafiart1ev1aaatCvAUfKttLearuWrP9MDH5MBPbIqV92AaeXatLxBI9gBaebbnrfifHhDYfgasaacPC6xNi=xH8viVGI8Gi=hEeeu0xXdbba9frFj0xb9qqpG0dXdb9aspeI8k8fiI+fsY=rqGqVepae9pg0db9vqaiVgFr0xfr=xfr=xc9adbaqaaeGacaGaaiaabeqaaeqabiWaaaGcbaWefv3ySLgznfgDOjdaryqr1ngBPrginfgDObcv39gaiqaacqWFecFrcqGGOaakcqWGAbGwdaWgaaWcbaGaeGymaedabeaakiabdQfaAnaaBaaaleaacqaIYaGmaeqaaOGaeiykaKIae8hHWx0aaSbaaSqaaGGaciab+17aUbqabaGccqGGBbWwcqWGfbqrcqGGOaakcqWGAbGwdaWgaaWcbaGaeGymaedabeaakiabcIcaOiab+17aUjabgkHiTiabdQfaAnaaBaaaleaacqaIXaqmaeqaaOGaeiykaKIaeiiFaWNae4xVd4Maeiyxa0Laeyypa0Jae8hHWx0aaSbaaSqaaiab+17aUbqabaGccqGGBbWwcqGF9oGBdaahaaWcbeqaaiabikdaYaaakiabdchaWjabgkHiTiab+17aUjabdchaWjabcIcaOiabigdaXiabgkHiTiabdchaWjabcMcaPiabgUcaRiab+17aUnaaCaaaleqabaGaeGOmaidaaOGaemiCaa3aaWbaaSqabeaacqaIYaGmaaGccqGGDbqxcqGH9aqpcqWGWbaCcqGGOaakcqaIXaqmcqGHsislcqWGWbaCcqGGPaqkcqGGOaakcqWFecFrdaWgaaWcbaGae4xVd4gabeaakiab+17aUnaaCaaaleqabaGaeGOmaidaaOGaeyOeI0Iae8hHWx0aaSbaaSqaaiab+17aUbqabaGccqGF9oGBcqGGPaqkaaa@8531@. Expressions for E(Z1),E(Z2),E(Z12)
 MathType@MTEF@5@5@+=feaafiart1ev1aaatCvAUfKttLearuWrP9MDH5MBPbIqV92AaeXatLxBI9gBaebbnrfifHhDYfgasaacPC6xNi=xH8viVGI8Gi=hEeeu0xXdbba9frFj0xb9qqpG0dXdb9aspeI8k8fiI+fsY=rqGqVepae9pg0db9vqaiVgFr0xfr=xfr=xc9adbaqaaeGacaGaaiaabeqaaeqabiWaaaGcbaWefv3ySLgznfgDOjdaryqr1ngBPrginfgDObcv39gaiqaacqWFecFrcqGGOaakcqWGAbGwdaWgaaWcbaGaeGymaedabeaakiabcMcaPiabcYcaSiab=ri8fjabcIcaOiabdQfaAnaaBaaaleaacqaIYaGmaeqaaOGaeiykaKIaeiilaWIae8hHWxKaeiikaGIaemOwaO1aa0baaSqaaiabigdaXaqaaiabikdaYaaakiabcMcaPaaa@4B23@ and E(Z22)
 MathType@MTEF@5@5@+=feaafiart1ev1aaatCvAUfKttLearuWrP9MDH5MBPbIqV92AaeXatLxBI9gBaebbnrfifHhDYfgasaacPC6xNi=xH8viVGI8Gi=hEeeu0xXdbba9frFj0xb9qqpG0dXdb9aspeI8k8fiI+fsY=rqGqVepae9pg0db9vqaiVgFr0xfr=xfr=xc9adbaqaaeGacaGaaiaabeqaaeqabiWaaaGcbaWefv3ySLgznfgDOjdaryqr1ngBPrginfgDObcv39gaiqaacqWFecFrcqGGOaakcqWGAbGwdaqhaaWcbaGaeGOmaidabaGaeGOmaidaaOGaeiykaKcaaa@3CBD@ are also easy to derive using this approach.

The authors' responses are provided in the text.

### Review 3 (W. Huber)

This reviewer provided no comments for publication.
